# Caffeine pollution changes anti-predator behaviour in *Gammarus pulex* similarly to manipulative parasites

**DOI:** 10.1098/rsbl.2026.0360

**Published:** 2026-09-09

**Authors:** Elias Fritzsche, Hannah Otto, Rudram Bhagwat, Nicole C. Bosse, Carola Mauel, Joachim G. Frommen, Timo Thünken

**Affiliations:** ^1^ Section Animal Biodiversity, Bonn Institute for Organismic Biology, University of Bonn, Bonn 53121, Germany; ^2^ School of Biological and Chemical Sciences, Manchester Metropolitan University, Manchester M1 5GD, UK

**Keywords:** host manipulation, anti-predator behaviour, environment dependence, coffee and tea, Acanthocephala, *Polymorphus minutus*, environmental change, anthropogenic effects

## Abstract

Caffeine is an emerging environmental contaminant in freshwater ecosystems that is primarily introduced through wastewater. Even at low concentrations, caffeine can affect the physiology and behaviour of aquatic organisms. Still, its ecological significance remains little understood. We examined the impact of environmentally relevant caffeine concentrations on the anti-predator behaviour of the freshwater amphipod *Gammarus pulex*. Gammarids play a central role in aquatic decomposition processes and are a primary food source for various aquatic predators. We examined the effects of caffeine in relation to a well-documented effect caused by the manipulative parasite *Polymorphus minutus*. This acanthocephalan parasite manipulates the anti-predator behaviour of its intermediate host, the gammarid, by altering its photophobia, thereby increasing its transmission to the final host, water birds. Using a two-choice experimental design, we assessed the phototactic behaviour of *P. minutus*-infected and uninfected *G. pulex* after 20 h of high- and low-concentration caffeine exposure. High concentrations of caffeine increased the activity of exposed individuals and reduced photophobia to a degree comparable with that of a *P. minutus* infection. These results suggest that human-derived chemicals can shift antipredator behaviour in a direction similar to an evolved parasite-mediated manipulation, which could increase predation risk and ultimately alter ecosystem dynamics.

## 1. Introduction

Freshwater ecosystems are facing drastic human-induced challenges, including increases in water temperature, turbidity or chemical contamination [1]. Caffeine is a widely used psychoactive compound that directly influences central nervous system processes [2]. Although caffeine is biodegradable under ideal conditions, it is often not fully eliminated by wastewater treatment processes [3–5]. Caffeine shows high stability with a half-life of 100 to 240 days, resulting in its persistence in aquatic systems [6,7]. Relevant caffeine levels have been reported globally in freshwater environments, from urban rivers to remote streams [8,9]. Likewise, caffeine accumulation has been documented in various marine and freshwater organisms [2,9,10]. Caffeine exposure has a wide range of physical and behavioural effects on aquatic species [11,12]. In fishes, it leads to changes in activity, aggression and anxiety-related behaviour [13–16]. In freshwater crustaceans, caffeine exposure causes reduced somatic growth and fecundity, as well as feeding inhibition [9,17]. It furthermore changes swimming speed and modifies movement patterns [18]. In addition to these sub-lethal effects, caffeine exposure can be lethal at higher concentrations [17]. There are rising concerns about the ecological impact of caffeine, as contamination has the potential to impair population dynamics of aquatic species, change ecosystem functioning and biodiversity [1]. In this context, understanding how caffeine affects fundamental ecological processes, such as predator–prey interactions and food-web dynamics, is of particular importance [19]. However, knowledge about the broader ecological impact of caffeine remains scarce.

Gammarids are amphipod crustaceans that play a vital role in aquatic ecosystems, serving as major decomposers of organic materials and a primary food source for numerous fish and bird species [20]. They are highly sensitive to chemical pollution, which may impact their ecological functions such as scavenging, herbivory and community structuring [21]. *Gammarus* species are photophobic, seeking shelter in dark areas to avoid predation, and reduce activity as part of their anti-predator behaviour [20,22–24]. They furthermore show high behavioural plasticity, for example, in response to the presence of predator cues [25]. Increased activity induced by psychoactive compounds such as caffeine has the potential to increase the risk of predation in gammarids [26,27].

Manipulative parasites may actively alter ecosystem dynamics by modifying activity and movement patterns of hosts, thereby influencing their temporal and spatial distribution as well as predation risk [28]. These changes in trophic interactions, food webs and eco-evolutionary feedbacks ultimately affect demographic processes in ecosystems [28]. Furthermore, parasites may function as both mitigators and amplifiers of pollution. Although parasites may reduce metal loads in host tissues, they can also exacerbate toxic effects by impairing host defence mechanisms and physiological balance [29–31].

Gammarids are intermediate hosts of acanthocephalan parasites, a group of endoparasites that feature a complex life cycle with an obligatory host change [32]. The infected gammarid needs to be ingested by a suitable vertebrate definitive (final) host [33], where the parasites reproduce [32]. To increase the chance of ingestion, the parasite alters the conspicuousness and behaviour of the intermediate host, ensuring the transmission of the parasite to the definitive host [32–36]. The acanthocephalan parasite *Polymorphus minutus* induces negative geotaxis, alters photophobic behaviour in its intermediate host [37–39] and increases drifting behaviour [40]. These behavioural changes increase the susceptibility of *G. pulex* to predation by water birds, the parasite's definitive hosts [22,24,41–44]. Hypoxia signalling has been hypothesized to contribute to the negative geotaxis of infected gammarids, which also displayed a decreased metabolic rate and increased brain lactate levels [38].

The effects of chemical pollution are often investigated in isolation and outside their ecological context. Here, we examine the effects of caffeine exposure on *Gammarus pulex* in the context of a well-documented natural factor known to alter gammarid behaviour: infection with the manipulative acanthocephalan parasite *P. minutus*. By incorporating this natural host–parasite interaction, our study provides a more ecologically realistic assessment of anthropogenic effects. We investigated how short-term caffeine exposure influences the activity and phototactic behaviour of *G. pulex*, and whether caffeine modulates *P. minutus*-induced behavioural alterations. We hypothesized that caffeine exposure may reduce photophobic behaviour owing to increased activity and amplify parasite-induced behavioural changes.

## 2. Material and methods

### (a) Experimental animals

*Gammarus pulex* were collected from a brook in Bonn, Germany (Dransdorfer Bach, 50° 44’ N, 7°02’ E), using hand nets. Individuals parasitized by the acanthocephalan *P. minutus* were recognizable by the bright orange coloration of the cystacanth stage visible through the gammarid cuticle [27]. Gammarids were transported in buckets with brook water to the laboratory at the Bonn Institute for Organismic Biology, University of Bonn.

### (b) Experimental set-up and procedure

In total, 120 gammarids were placed in 12 tanks (30 cm × 20 cm × 20 cm), each filled with 5 l of water taken from the natural habitat. Each tank was aerated by an air stone and contained a single dead leaf taken from the brook as a food resource and shelter for the test individuals. The tanks were arranged in an alternating order of high and low caffeine concentrations (see below) to minimize confounding effects by external factors. Ten gammarids were placed in each tank, five *P. minutus*-infected and five uninfected individuals. The gammarids were exposed to the caffeine treatment for 20 h before the experiment commenced. The experimental room was illuminated with a ceiling lamp (Radium Bonalux Super NL 54 W/840 white), which was switched on during the phototaxis trials. Additionally, indirect natural light could enter through a transom window, ensuring a natural light–dark rhythm typical of mid-July. Light intensities during the behavioural observations varied approximately between 300 and 500 lx.

A master caffeine solution was prepared by dissolving 0.6 mg of pure caffeine (CO750-5G, Sigma-Aldrich) in 10 l of tap water, resulting in a concentration of 60000 ng l^–1^. For the high-concentration treatment, 1 l of the master solution was added to six of the tanks, achieving a final concentration of 10 000 ng l^–1^. This concentration has been detected in highly polluted rivers [8]. For the low-concentration treatment, 10 ml of the master solution was diluted with 990 ml of tap water and added to the remaining six tanks, resulting in a final concentration of 100 ng l^–1^. The water in the 12 tanks was not changed during the 20-h period.

For the experiment, a transparent plastic tube (25.5 cm length and 4 cm diameter) was divided into a light and a dark half. To create a dark area, the half of the tube was wrapped with black tape. The tube was fixed with a thin thread on a green styrofoam plate and filled to a height of 2 cm with brook water that was kept in an additional tank. Before each trial, the water in the tube was changed, and the water temperature was measured. It ranged between 16.0 and 16.6°C. A single gammarid was carefully put into the tube through a small hole in the middle using a pipette. After 2 min of acclimation, the gammarid was observed for a period of 5 min. To estimate phototactic behaviour, we measured the amount of time spent by the test individuals on the light side (tl) using a stopwatch. Time on the dark side (td) was calculated by subtracting tl from the 300 s total observation time. Furthermore, we counted how often the test animal switched between the light side and the dark side as a proxy for activity [45]. Between each trial, the tube was rotated by 180° to control for potential side effects. After each trial, the gammarids were sedated using sparkling water, and their length was measured using a digital calliper in a relaxed position (*N* = 100, mean (mm) ± s.d.: 7.55 ± 1.4).

In total, we conducted 100 trials: 54 using infected individuals (28 high caffeine, 26 low caffeine) and 46 using uninfected individuals (23 high caffeine, 23 low caffeine). The remaining 20 individuals served as reserve and were not used in the study. For each trial, the choice between individuals coming from high or low concentration tanks and between infected and uninfected individuals was made haphazardly. The observer was blinded to the treatment conditions. We acknowledge that the tank of origin was not recorded for individual organisms during behavioural testing. Consequently, it was not possible to include tank as a random effect in the statistical analyses. Nevertheless, because each caffeine treatment was replicated across six independent tanks and animals were haphazardly sampled, we consider it unlikely that the observed treatment effects were driven solely by tank-specific conditions, although we cannot completely exclude some degree of pseudoreplication.

### (c) Statistical analysis

Out of the 100 trials, two trials were excluded from the analysis owing to inactivity of the gammarids during the entire experiment (both were infected gammarids coming from the low caffeine concentration). To analyse phototaxis, the relative time at the light side was calculated using the following formula:

$$\begin{array}{r} Preference\;index=\left(tl-td\right)/\left(tl+td\right) \end{array}$$

*tl* = time spent on the light side

*td* = time spent on the dark side

Hence, positive values indicate photophilia and negative values indicate photophobia. Data were analysed using R (version 4.4.0 [46]). The effects of caffeine exposure (high/low concentration) and infection status (infected/uninfected), as well as the interactive effect of both factors on phototactic behaviour, were analysed by fitting a linear model (LM). The preference index was log-transformed to achieve normality of residuals (see supplementary material). The effects of caffeine exposure, infection status and their interaction on activity were analysed using a generalized LM (GLM). Owing to over-dispersion, a quasi-Poisson error distribution was applied (see supplementary material). Interactive effects between explanatory variables were tested using model comparisons. Non-significant interaction terms were removed [47]. Test statistics are reported for the final models as well as for the individual predictors (see supplementary material). Effect sizes were calculated as partial Eta^2^ for the LM and incidence rate ratio (IRR) for the GLM using the R package effectsize [48].

## 3. Results

**Figure 1.**
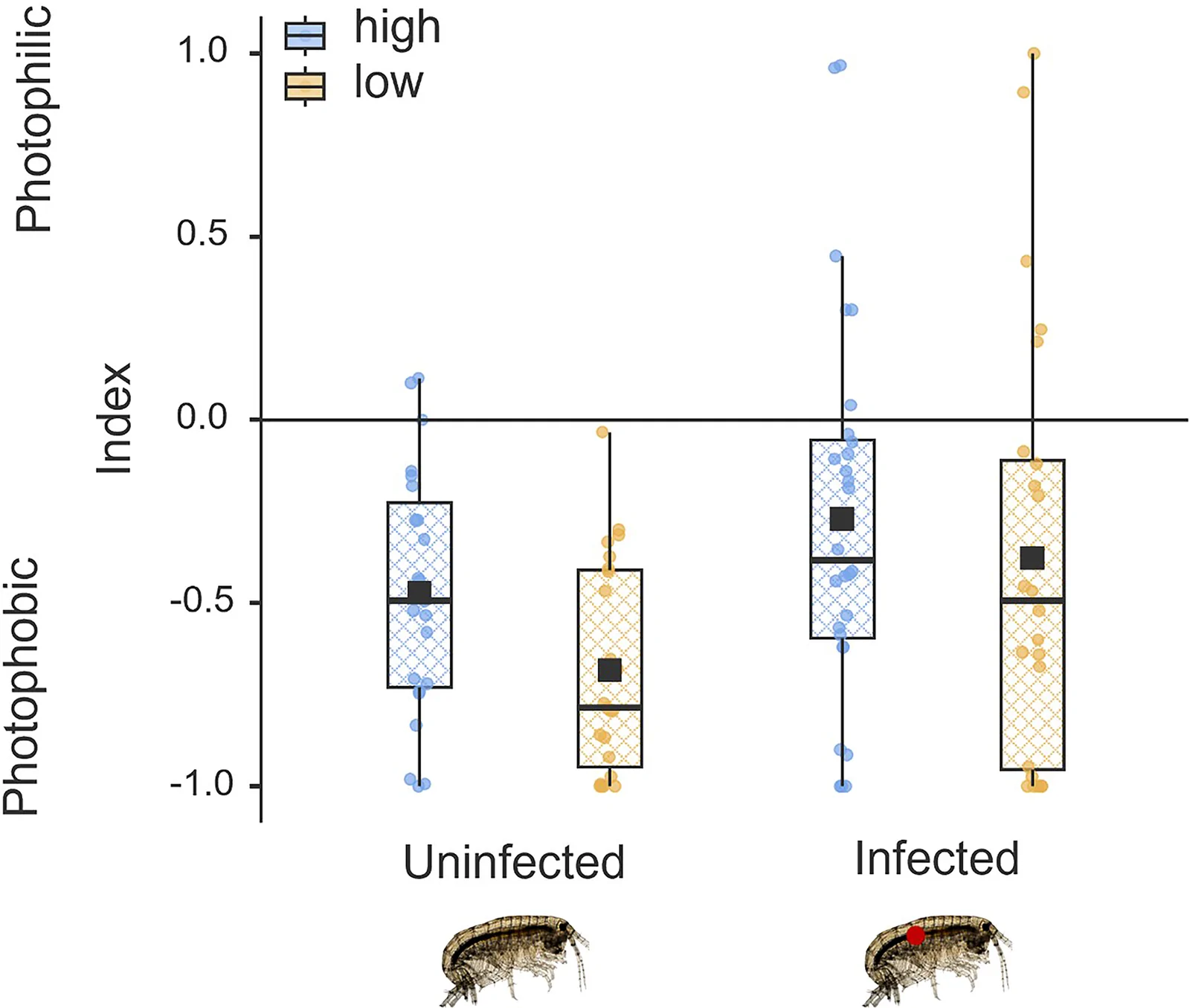
Phototaxis of *P. minutus*-infected and uninfected *G. pulex* in relation to caffeine pollution (high concentration/low concentration). Shown is the relative time at the dark or light side. Positive values indicate photophilia and negative values photophobia. Mean values are shown as black squares. Boxes represent first and third quartiles, bold lines medians and whiskers the last values within the 1.5-fold interquartile range. Individual data points are shown as dots.

**Figure 2.**
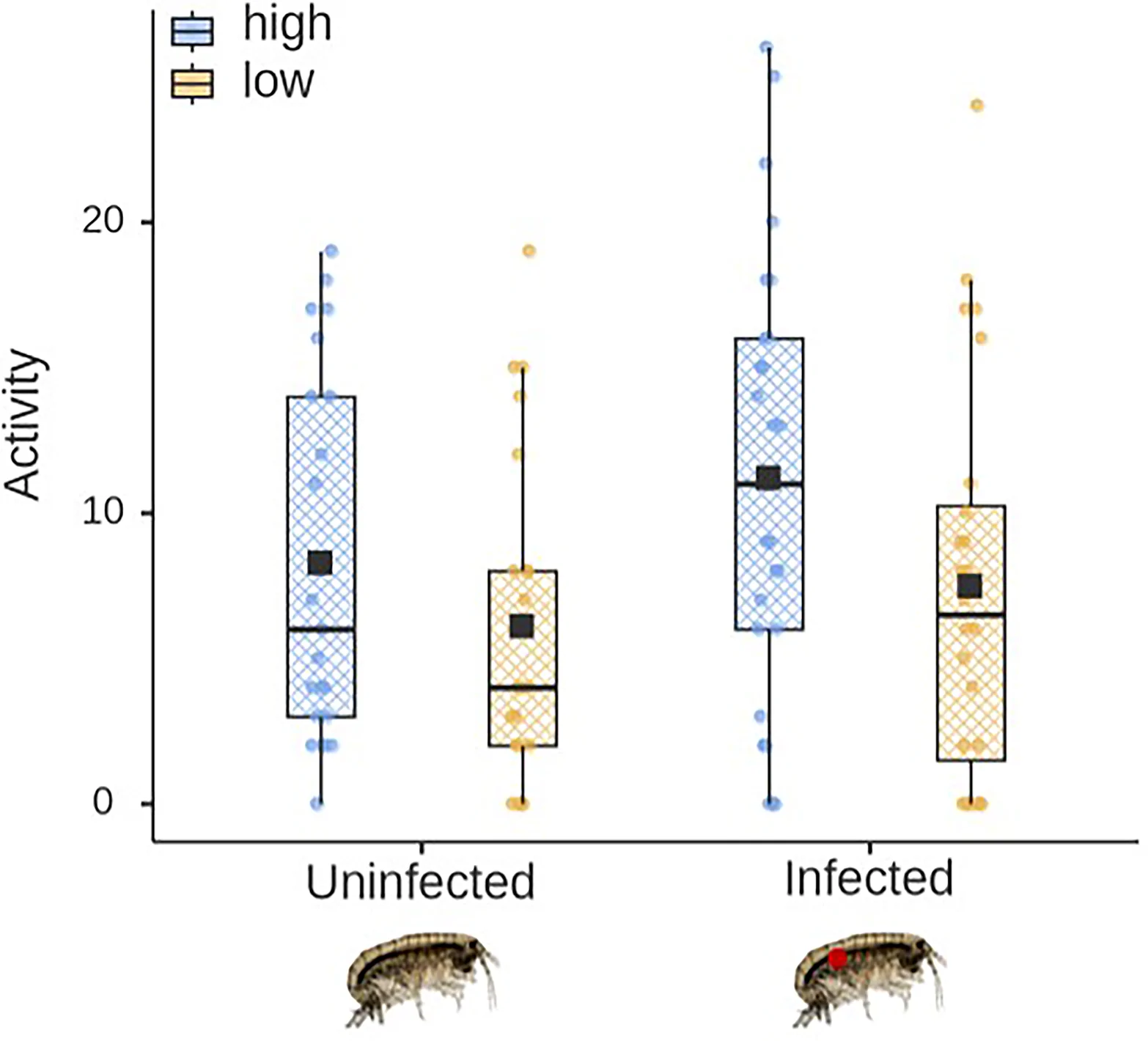
Activity (changes between dark and light side) of *P. minutus*-infected and uninfected *G. pulex* in relation to the caffeine pollution (high concentration/low concentration). Mean values are shown as black squares. Boxes represent first and third quartiles, bold lines medians and whiskers the last values within the 1.5-fold interquartile range. Individual data points are shown as dots.

First, we tested whether exposure to high caffeine concentrations altered the effect of the *P. minutus* infection. This was not the case, as the interactions between infection and caffeine exposure were neither significant for phototaxis (d.f. = 1, *F* = 0.361, *p* = 0.550, figure 1) nor for activity (d.f. = 1, *F* = 0.089, *p* = 0.767; figure 2). The interaction term was therefore removed from both models. Final models were both significant (phototaxis: *F*_2,95_ = 4.863, *p* = 0.001; activity: *F*_2,95_ = 4.016, *p* = 0.021). Caffeine exposure and infection with *P. minutus* independently affected the behaviour of *G. pulex*. Gammarids exposed to high caffeine concentrations were less photophobic (*p* = 0.041; table 1; figure 1) and showed higher activity, that is, they switched more often between the light and dark side (*p* = 0.028; table 2; figure 2) than gammarids exposed to low caffeine concentrations. Parasite-infected gammarids were less photophobic than uninfected ones (*p* = 0.026; table 1; figure 1), but both infection groups did not significantly differ in activity (*p* = 0.10; table 2; figure 2). The effect sizes were comparable for caffeine exposure and infection status, particularly for phototaxis (tables 1 and 2).

**Table 1.** Results of the LM with the index (log) as the dependent variable.

dependent variable	explanatory variable	estimate	SE	*t*	*p*	partial Eta²	95% CI
phototaxis (log)	caffeine (low vs. high)	–0.178	0.086	-2.070	**0.041**	0.05	–0.349, –0.007
	infection status (inf. vs. uninf.)	0.194	0.086	2.251	**0.026**	0.05	0.022, 0.365

NOTE: The explanatory variables are the infection status and caffeine concentration.

**Table 2.** Results of the GLM with the number of crossed lines as the dependent variable.

dependent variable	explanatory variable	estimate	SE	*t*	*p*	IRR	95% CI
activity	infection status (inf. vs. uninf.)	0.2630	0.1623	1.621	0.108	1.300	0.948, 1.795
	caffeine (low vs. high)	–0.3638	0.1633	–2.228	**0.028**	0.695	0.502, 0.954

NOTE: The explanatory variables are the infection status and caffeine concentration. IRR and 95% confidence intervals.

## 4. Discussion

The aim of this study was to investigate whether caffeine pollution in freshwater ecosystems affects the anti-predator behaviour of *G. pulex*. The effects of caffeine exposure were examined in relation to behavioural alterations induced by the manipulative parasite *P. minutus* to obtain a more realistic assessment of the ecological relevance of caffeine pollution. Gammarids exposed to high caffeine concentrations were less photophobic and showed higher activity, that is, they switched more often between the light and dark side than gammarids of the low concentration group. These results are in accordance with findings in other aquatic species highlighting that caffeine exposure affects behaviour [12,13,18,49].

In crustaceans, caffeine exposure has been associated with increased locomotor activity and altered swimming patterns in copepods [18], whereas *Daphnia magna* shows more variable responses that can become severe at higher concentrations or prolonged exposure, including impaired swimming, feeding inhibition and mortality [9,11]. Similar dose- and time-dependent effects have been reported in fishes, where short-term exposure may increase activity, whereas longer exposure can lead to developmental abnormalities and lethality [13,15,16,50]. Together, these studies indicate that caffeine can disrupt neural and behavioural processes in aquatic organisms, although the magnitude and direction of effects are context-dependent [13,15].

Infection with *P. minutus* reduced the photophobic behaviour of the *Gammarus* host but did not affect its activity. Comparisons of effect sizes revealed that the impact of caffeine exposure on phototaxis was comparable with that of a natural *P. minutus* infection. Although both effects were rather small, they could nevertheless be ecologically important, particularly because gammarids often occur at high population densities and play key roles in aquatic ecosystems, thereby influencing ecosystem processes such as decomposition, nutrient recycling and trophic energy flow. Recent research has highlighted that even subtle effects of manipulative parasites can have important ecological consequences by altering predator–prey dynamics and, consequently, food-web structure [42,51]. The ecological impact of caffeine pollution needs to be analysed in future studies. We expect that the increased predation risk associated with reduced photophobia and elevated activity will decrease gammarid population sizes. Such reductions could strongly affect ecosystem functions provided by gammarids within aquatic communities, including leaf-litter shredding and their role as prey for vertebrate and invertebrate predators, while also reducing their impact as predators of other invertebrates [52].

In the present study, we did not detect any significant interactive effects between parasitism and caffeine exposure on phototactic or activity behaviour, indicating that caffeine neither enhanced nor reduced the manipulative effect of the parasite. In other words, the effects of caffeine exposure and parasite infection did not add up or cancel each other out. However, we assume that reduced photophobia and elevated activity of gammarids in caffeine-polluted habitats may increase the general predation risk for both infected and uninfected individuals, thereby potentially affecting parasite transmission success to the definitive host. Such changes may, in turn, alter the selection pressure on both the parasite's need to manipulate host behaviour and the host's resistance to this manipulation. The strength and direction of these selective pressures are probably dependent on demographic and ecological factors such as gammarid population density, predator community composition and parasite prevalence [53]. Nevertheless, our results indicate that caffeine pollution alters gammarids’ antipredator behaviour, which has the potential to modify complex host–parasite interactions. The specific ecological and evolutionary consequences of these effects remain to be investigated in future studies.

The inclusion of a caffeine-free control would have allowed a direct assessment of baseline behavioural responses to caffeine. However, our study was designed to compare two environmentally relevant caffeine concentrations representing common background contamination (low concentration) and a highly polluted scenario (high concentration). Consequently, the present results should be interpreted as evidence for concentration-dependent effects of caffeine rather than for the effects of caffeine exposure *per se*. The observed behavioural differences indicate that elevated caffeine concentrations can significantly alter gammarid behaviour, including responses that resemble parasite-induced manipulation.

Overall, our findings highlight that an environmental contaminant can shift antipredator behaviour in an ecologically important freshwater species in a direction similar to an evolved parasite-mediated manipulation. This suggests that chemical pollution may act as an additional driver of behaviourally mediated ecological interactions [10,20], with potential consequences for predator–prey dynamics, food-web structure and ecosystem functioning in freshwater systems [28,51].

## Supplementary Material



## Data Availability

Supplementary material is available online [54].

## References

[1] Nunes B . 2014 Pharmaceutical drugs and other substances with pharmacological activity in the environment: a threat to biodiversity? Conserv. Sci. 2, 12–16. (doi:10.3126/cs.v2i1.13764)

[2] Vieira LR , Soares AMVM, Freitas R. 2022 Caffeine as a contaminant of concern: a review on concentrations and impacts in marine coastal systems. Chemosphere 286, 131675. (doi:10.1016/j.chemosphere.2021.131675) 34358890

[3] Rigueto CVT , Nazari MT, De Souza CF, Cadore JS, Brião VB, Piccin JS. 2020 Alternative techniques for caffeine removal from wastewater: an overview of opportunities and challenges. J. Water Process Eng. 35, 101231. (doi:10.1016/j.jwpe.2020.101231)

[4] Raj R , Tripathi A, Das S, Ghangrekar MM. 2021 Removal of caffeine from wastewater using electrochemical advanced oxidation process: a mini review. Case Stud. Chem. Environ. Eng. 4, 100129. (doi:10.1016/j.cscee.2021.100129)

[5] Korekar G , Kumar A, Ugale C. 2020 Occurrence, fate, persistence, and remediation of caffeine: a review. Environ. Sci. Pollut. Res. 27, 34715–34733. (doi:10.1007/s11356-019-06998-8) 31811612

[6] Hillebrand O , Nödler K, Licha T, Sauter M, Geyer T. 2012 Caffeine as an indicator for the quantification of untreated wastewater in karst systems. Water Res. 46, 395–402. (doi:10.1016/j.watres.2011.11.003) 22104295

[7] Tawfik NA , El-Bakary ZA, Abd El-Wakeil KF. 2024 Determination of caffeine in treated wastewater discharged in the Nile River with emphasis on the effect of zinc and physicochemical factors. Environ. Sci. Pollut. Res. 31, 28124–28138. (doi:10.1007/s11356-024-32918-6) PMC11058622 38530524

[8] Li S , Wen J, He B, Wang J, Hu X, Liu J. 2020a Occurrence of caffeine in the freshwater environment: implications for ecopharmacovigilance. Environ. Pollut. 263, 114371. (doi:10.1016/j.envpol.2020.114371) 32217417

[9] Rodrigues S , Alves RS, Antunes SC. 2025 Impact of caffeine on aquatic ecosystems: assessing trophic-level biological responses. J. Xenobiot. 15, 86. (doi:10.3390/jox15030086) 40558869 PMC12193926

[10] Ondarza PM , Haddad SP, Avigliano E, Miglioranza KS, Brooks BW. 2019 Pharmaceuticals, illicit drugs and their metabolites in fish from Argentina: implications for protected areas influenced by urbanization. Sci. Total Environ. 649, 1029–1037. (doi:10.1016/j.scitotenv.2018.08.383) 30308876

[11] Nunes B , Santos J, Dionísio R, Dias de Alkimin G. 2022 Investigation of potential behavioral and physiological effects of caffeine on *D. magna*. Environ. Sci. Pollut. Res. 29, 43237–43250. (doi:10.1007/s11356-022-18695-0) 35094280

[12] Santos N , Picolo V, Domingues I, Perillo V, Villacis RA, Grisolia CK, Oliveira M. 2023 Effects of environmental concentrations of caffeine on adult zebrafish behaviour: a short-term exposure scenario. Environ. Sci. Pollut. Res. 30, 63776–63787. (doi:10.1007/s11356-023-26799-4) PMC10172215 37058238

[13] Aliko V , Mehmeti E, Qirjo M, Faggio C. 2019 ‘Drink and sleep like a fish’: goldfish as a behavior model to study pharmaceutical effects in freshwater ecosystems. J. Biol. Res. 92, 7939. (doi:10.4081/jbr.2019.7939)

[14] Bikker J , MacDougall-Shackleton H, Bragg LM, Servos MR, Wong BBM, Balshine S. 2024 Impacts of caffeine on fathead minnow behaviour and physiology. Aquat. Toxicol. 273, 106982. (doi:10.1016/j.aquatox.2024.106982) 38861791

[15] Dos Santos JA *et al.* 2022 Sublethal effects of environmental concentrations of caffeine on a neotropical freshwater fish. Ecotoxicology 31, 161–167. 34773559 10.1007/s10646-021-02498-z

[16] Moore MT , Greenway SL, Farris JL, Guerra B. 2008 Assessing caffeine as an emerging environmental concern using conventional approaches. Arch. Environ. Contam. Toxicol. 54, 31–35. (doi:10.1007/s00244-007-9059-4) 17957400

[17] Li S , He B, Wang J, Liu J, Hu X. 2020b Risks of caffeine residues in the environment: necessity for a targeted ecopharmacovigilance program. Chemosphere 243, 125343. (doi:10.1016/j.chemosphere.2019.125343) 31751929

[18] Moreno-Vega G , Frazão LR, De-La-Cruz LT, Lopes RM. 2025 Changes in the swimming behavior of *Temora turbinata* (Copepoda, Calanoida) in response to sub-lethal concentrations of caffeine and triclosan. Aquat. Toxicol. 283, 107352. (doi:10.1016/j.aquatox.2025.107352) 40209295

[19] Mustard JA . 2014 The buzz on caffeine in invertebrates: effects on behavior and molecular mechanisms. Cell. Mol. Life Sci. 71, 1375–82. (doi:10.1007/s00018-013-1497-8) 24162934 PMC3961528

[20] Bollache L , Kaldonski N, Troussard JP, Lagrue C, Rigaud T. 2006 Spines and behaviour as defences against fish predators in an invasive freshwater amphipod. Anim. Behav. 72, 627–633. (doi:10.1016/j.anbehav.2005.11.020)

[21] Conlan KE . 1994 Amphipod crustaceans and environmental disturbance: a review. J. Nat. Hist. 28, 519–554. (doi:10.1080/00222939400770241)

[22] Bethel WM , Holmes JC. 1973 Altered evasive behavior and responses to light in amphipods harboring acanthocephalan cystacanths. J. Parasitol. 59, 945–956. (doi:10.2307/3278623) 4821113

[23] Dudley Williams D , Moore KA. 1982 The effect of environmental factors on the activity of *Gammarus pseudolimnaeus* (Amphipoda). Hydrobiologia 96, 137–147. (doi:10.1007/BF02185429)

[24] Thünken T , Vitt S, Baldauf SA, Jung T, Frommen JG 2018 Individual behavioural responses of an intermediate host to a manipulative acanthocephalan parasite and the effects of intra-specific parasite competition. Evol. Ecol. Res. 19, 503–516.

[25] Kullmann H , Thünken T, Baldauf SA, Bakker TCM, Frommen JG. 2008 Fish odour triggers conspecific attraction behaviour in an aquatic invertebrate. Biol. Lett. 4, 458–460. (doi:10.1098/rsbl.2008.0246) 18593668 PMC2610077

[26] Wooster DR . 1998 Amphipod (*Gammarus minus*) responses to predators and predator impact on amphipod density. Oecologia 115, 253–259. (doi:10.1007/s004420050514) 28308459

[27] Thünken T , Baldauf SA, Bersau N, Bakker TCM, Kullmann H, Frommen JG. 2010 Impact of olfactory non-host predator cues on aggregation behaviour and activity in *Po**l**ymorphus minutus* infected *Gammarus pulex*. Hydrobiologia 654, 137–145. (doi:10.1007/s10750-010-0377-6)

[28] Lefèvre T , Lebarbenchon C, Gauthier-Clerc M, Missé D, Poulin R, Thomas F. 2009 The ecological significance of manipulative parasites. Trends Ecol. Evol. 24, 41–48. (doi:10.1016/j.tree.2008.08.007) 19026461

[29] Malek M , Haseli M, Mobedi I, Ganjali MR, MacKenzie K. 2007 Parasites as heavy metal bioindicators in the shark *Carcharhinus dussumieri* from the Persian Gulf. Parasitology 134, 1053–1056. (doi:10.1017/S0031182007002508) 17326849

[30] Sures B . 2008 Host–parasite interactions in polluted environments. J. Fish Biol. 73, 2133–2142. (doi:10.1111/j.1095-8649.2008.02057.x)

[31] Mehana ESE , Khafaga AF, Elblehi SS, Abd El-Hack ME, Naiel MA, Bin-Jumah M, Othman SI, Allam AA. 2020 Biomonitoring of heavy metal pollution using acanthocephalans parasite in ecosystem: an updated overview. Animals 10, 811. (doi:10.3390/ani10050811) 32392878 PMC7278602

[32] Bakker TCM , Frommen JG, Thünken T. 2017 Adaptive parasitic manipulation as exemplified by acanthocephalans. Ethology 123, 779–784. (doi:10.1111/eth.12660)

[33] Thünken T , Baldauf SA, Bersau N, Frommen JG, Bakker TCM. 2019 Parasite-induced colour alteration of intermediate hosts increases ingestion by suitable final host species. Behaviour 156, 1329–1348.(doi:10.1163/1568539x-00003568)

[34] Baldauf SA , Thünken T, Frommen JG, Bakker TCM, Heupel O, Kullmann H. 2007 Infection with an acanthocephalan manipulates an amphipod's reaction to a fish predator's odours. Int. J. Parasitol. 37, 61–65. (doi:10.1016/j.ijpara.2006.09.003) 17049528

[35] Poulin R . 2013 Parasite manipulation of host personality and behavioural syndromes. J. Exp. Biol. 216, 18–26. (doi:10.1242/jeb.073353) 23225863

[36] Perrot-Minnot MJ *et al.* 2023 Hooking the scientific community on thorny-headed worms: interesting and exciting facts, knowledge gaps and perspectives for research directions on Acanthocephala. Parasite 30, 23. (doi:10.1051/parasite/2023026) 37350678 PMC10288976

[37] Bauer A , Haine ER, Perrot-Minnot MJ, Rigaud T. 2005 The acanthocephalan parasite *Polymorphus minutus* alters the geotactic and clinging behaviours of two sympatric amphipod hosts: the native *Gammarus pulex* and the invasive *Gammarus roeseli*. J. Zool. 267, 39–43. (doi:10.1017/S0952836905007223)

[38] Perrot-Minnot MJ , Maddaleno M, Cézilly F. 2016 Parasite-induced inversion of geotaxis in a freshwater amphipod: a role for anaerobic metabolism? Funct. Ecol. 30, 780–788. (doi:10.1111/1365-2435.12516)

[39] Bailly Y , Cézilly F, Rigaud T. 2018 Stage-dependent behavioural changes but early castration induced by the acanthocephalan parasite *Polymorphus minutus* in its *Gammarus pulex* intermediate host. Parasitology 145, 260–268. (doi:10.1017/S0031182017001457) 28831959

[40] McCahon CP , Maund SJ, Poulton MJ. 1991 The effect of the acanthocephalan parasite (*Pomphorhynchus laevis*) on the drift of its intermediate host (*Gammarus pulex*). Freshw. Biol. 25, 507–513. (doi:10.1111/j.1365-2427.1991.tb01393.x)

[41] Franceschi N , Rigaud T, Moret Y, Hervant F, Bollache L. 2007 Behavioural and physiological effects of the trophically transmitted cestode parasite, *Cyathocephalus truncatus*, on its intermediate host, *Gammarus pulex*. Parasitology 134, 1839–1847. (doi:10.1017/S0031182007003228) 17640401

[42] Giari L , Fano EA, Castaldelli G, Grabner D, Sures B. 2020 The ecological importance of amphipod–parasite associations for aquatic ecosystems. Water 12, 2429. (doi:10.3390/w12092429)

[43] Williams MA , Holland CV, Donohue I. 2020 Warming can alter host behavior in a similar manner to infection with behavior-manipulating parasites. Oecologia 194, 65–67. (doi:10.1007/s00442-020-04745-2) 32876762

[44] Fanton H , Franquet E, Logez M, Kaldonski N. 2021 Effects of temperature and a manipulative parasite on the swimming behaviour of *Gammarus pulex* in flowing water. Hydrobiologia 848, 4467–4476. (doi:10.1007/s10750-021-04655-1)

[45] Amin P , Frommen JG, Newton-Youens J. 2026 The effect of background colour on behaviour and development of golden mantella (*Mantella aurantiaca*) tadpoles. Ethology 132, 204–215. (doi:10.1111/eth.70046)

[46] R Core Team . 2013 R: a language and environment for statistical computing. Vienna, Austria: R Foundation for Statistical Computing.

[47] Engqvist L . 2005 The mistreatment of covariate interaction terms in linear model analyses of behavioural and evolutionary ecology studies. Anim. Behav. 70, 967–971. (doi:10.1016/j.anbehav.2005.01.016)

[48] Ben-Shachar MS , Lüdecke D, Makowski D. 2020 effectsize: estimation of effect size indices and standardized parameters. J. Open Source Softw. 5, 2815. (doi:10.21105/joss.02815)

[49] Ruiz-Oliveira J , Silva PF, Luchiari AC. 2019 Coffee time: low caffeine dose promotes attention and focus in zebrafish. Learn. Behav. 47, 227–233. (doi:10.3758/s13420-018-0369-3) 30623296

[50] Rodriguez RS , Haugen R, Rueber A, Huang CC. 2014 Reversible neuronal and muscular toxicity of caffeine in developing vertebrates. Comp. Biochem. Physiol. C Toxicol. Pharmacol. 163, 47–54. (doi:10.1016/j.cbpc.2014.03.004) 24667760

[51] Klemme I , Perälä T, Lehtinen SO, Kuparinen A. 2024 Parasite-mediated changes in host traits alter food web dynamics. Oikos 2024, e10374. (doi:10.1111/oik.10374)

[52] MacNeil C , Dick JT, Elwood RW. 1997 The trophic ecology of freshwater *Gammarus* spp. (Crustacea: Amphipoda): problems and perspectives concerning the functional feeding group concept. Biol. Rev. 72, 349–364. (doi:10.1017/s0006323196005038)

[53] Poulin R . 2014 Parasite biodiversity revisited: frontiers and constraints. Int. J. Parasitol. 44, 581–589. (doi:10.1016/j.ijpara.2014.02.003) 24607559

[54] Fritzsche E , Otto H, Bhagwat R, Bosse NC, Mauel C, Frommen JG, Thünken T. 2026 Supplementary material from: Caffeine pollution changes anti-predator behaviour in *Gammarus pulex* similarly to manipulative parasites. Figshare. (doi:10.6084/m9.figshare.c.8627738) PMC13561076 42710883

