# Supplementary material for: Caffeine pollution changes anti-predator behaviour in Gammarus pulex similarly to manipulative parasites
Source: Biol Lett. 2026 Sep 9;22(9):20260360. doi: 10.1098/rsbl.2026.0360 (PMC13561076; doi:10.1098/rsbl.2026.0360)
Supplement: Supplementary file 1 [file rsbl.2026.0360.zip › rsbl.2026.0360/rsbl20260360_si_002.docx]

**Supplemental material to Fritzsche et al. “Caffeine pollution changes antipredator behaviour in *Gammarus pulex* similarly to manipulative parasites*”***

**Statistical analyses**

Phototaxis

The effects of infection status (infected vs. uninfected), caffeine exposure (high vs. low concentration), and their interaction on phototaxis (index) were analysed using a linear model (LM, index ~ infection * caffeine). Model residuals were assessed for normality using the check_normality function from the R package performance (Lüdecke et al. 2021). As residuals deviated significantly from normality (p < 0.001), the response variable was log-transformed. Because the logarithm is defined only for positive values (Sokal & Rohlf, 19987), a constant of 1.5 was added to the index (which ranges from -1 to 1) prior to transformation. Residuals of the transformed model did not deviate significantly from normality (p = 0.163). In addition, residuals were visually inspected for potential deviations from a normal distribution (see Fig. S1).

The significance of the interaction term was assessed by comparing nested models using analysis of variance (F-tests; Model 1: TransIndex ~ Infection * Caffeine; Model 2: TransIndex ~ Infection + Caffeine) using the anova () function in R. As the interaction term was not significant (see results), it was removed from the model. The final model (TransIndex ~ Infection + Caffeine) was evaluated against a null model using an F-test. The F-statistic for overall model fit was obtained from the model summary, while significance of individual predictors was assessed based on the corresponding t-tests. Additionally, non-parametric Wilcoxon rank-sum tests based on the original (untransformed) index data were conducted as a robustness check, yielding consistent results. Significant effects were observed for both caffeine (W = 1506, p = 0.029) and infection (W = 905, p = 0.038).

Fig. S1: QQplots of the transformed model examine the effects of caffeine concentration and *Polymorphus minutus* infection on phototaxis.

Activity

Activity was measured as count data that were overdispersed according to the check_overdispersion function from the R package performance (dispersion ratio = 5.249, Pearson’s χ² = 493.437, p < 0.001). The effects of infection status (infected vs. uninfected), caffeine exposure (high vs. low concentration), and their interaction on activity were therefore analysed by fitting a generalized linear model with a quasi-Poisson error distribution (GLM; activity ~ caffeine* infection, family = quasipoisson(link = "log")).

The significance of the interaction term was assessed by comparing nested models using analysis of variance (F-tests; Model 1: activity ~ Infection * Caffeine; Model 2: activity ~ Infection + Caffeine). As the interaction was not significant, it was removed from the model (see results). The final model (activity ~ Infection + Caffeine) was compared to a null model (activity ~ 1) using an F-test based on analysis of variance. The significance of individual predictors was obtained from t-tests reported in the model summary. Robustness checks using Wilcoxon rank-sum tests yielded results consistent with the GLM. A significant effect was found for caffeine (W = 1505, p = 0.029), whereas no significant effect was detected for infection (W = 996.5, p = 0.155).

**References**

Lüdecke, D., Ben-Shachar, M. S., Patil, I., Waggoner, P., & Makowski, D. (2021). performance: An R package for assessment, comparison and testing of statistical models. Journal of Open Source Software, 6, 3139

Sokal, R. R., & Rohlf, F. J. (1987). Biostatistics. Francise & Co, New York
